# Authentic Pathology Specimen Reception: A Valuable Resource for Developing Biomedical Science Student Competencies and Employability

**DOI:** 10.3389/bjbs.2023.11731

**Published:** 2023-09-25

**Authors:** T. Hussain, S. Namvar, M. Jones

**Affiliations:** ^1^ School of Science, Engineering and Environment, University of Salford, Manchester, United Kingdom; ^2^ Biomedical Research Centre, School of Science, Engineering and Environment, University of Salford, Manchester, United Kingdom; ^3^ Faculty of Biology Medicine and Health, School of Biological Sciences, Manchester Academic Health Science Centre, University of Manchester, Manchester, United Kingdom

**Keywords:** biomedical science education, specimen reception simulation, authentic education, graduate capital, employability

## Abstract

**Background/Introduction:** The pathology specimen reception is fundamental to the services provided by Biomedical Science laboratories worldwide. To ensure patient safety and that samples are of adequate quality to send for analysis, prospective Biomedical Scientists should have a robust knowledge of the processes involved and the acceptance criteria of the pathology specimen reception. This knowledge has been highlighted by employers as a current gap in Biomedical Science graduates and therefore needs to be addressed within higher education settings. To do this, this study aimed to 1) design a practical session to simulate the key processes of the pathology specimen reception and 2) to understand Biomedical Science students’ opinions on these activities and the development of transferable skills required for post-graduate employment.

**Methods:** The practical session was designed based on industrial requirements and academic knowledge of student skill sets to ensure suitability. Qualitative information regarding participant demographics and career interests was acquired through open-answer or multiple-choice questions. Quantitative student feedback was acquired via questionnaires utilising a 5-point Likert scale (*n* = 77).

**Results:** The scenario-based practical session provided students with a positive learning experience with 98.7% of participants enjoying the session, with 87.0% stating they learned a lot by completing the session. It was also identified that participants preferred this style of learning to that of conventional higher education teaching modalities with 97.4% stating they would prefer simulated employment focussed scenarios embedded into the curriculum more often. The majority of participants also thought this session was helpful for the development of their key transferrable skills including teamworking, communication, and confidence. When stratified based on demographic data, there was minimal difference between cohorts and in the majority of cases, those participants from non-traditional university entry backgrounds had a more positive experience and better transferable skill development following the completion of this style of learning experience.

**Conclusion:** This study highlights simulation-based learning as a tool to develop core Biomedical Science knowledge, build student graduate capital, and ensure the preparedness of students for post-graduation employment.

## Introduction

The Pathology laboratory specimen reception, well known as Central Specimen Reception (CSR) in many Pathology laboratories, is fundamental to the analytical processes and services provided by the Pathology department. All sample handling, preparation and data entry is managed within CSR; limitations in the operation of these processes will impact the integrity of the procedures performed further down the process. Specimen reception duties are mainly carried out by medical laboratory assistants (MLA) who play an integral role in Pathology laboratory services.

With patient safety being paramount in all areas of pathology, the CSR is integral at every point of the testing pathway. All healthcare professionals have a duty of care and a legal obligation to ensure that all the steps in the sample journey from collection to the final test results are processed correctly in line with the guidelines to guarantee that all results from processed samples match the patient [1]. Samples received at CSR are first checked to ensure they meet the sample acceptance criteria. This process involves ensuring that the sample has been correctly labelled with the patient details and match those on the request form, checking the sample has been collected in the correct tube and ensuring there is a sufficient volume of sample to meet the test requirements. The sample and test request form are then labelled with a unique barcode number to allow test results to be traced back to the patient. Samples are then prepared for analysis depending on the type of test requested.

Negligence during this process can result in the incorrect reporting of results with detrimental outcomes for the patient, with pre-analytical errors during this process accounting for more than two-thirds of all reported laboratory errors [2]. This demonstrates the importance of CSR within the pathology pipeline as indicated by the Institute of Biomedical Science (IBMS) [3]. Staff who work on the CSR require high levels of accuracy and attention to detail whist maintaining appropriate records and documentation [4]. Biomedical Scientists rely on all checks and sample preparation processes being carried out correctly on CSR prior to validating and authorising test results once samples have been processed. It is therefore desired that before entering employment, prospective applicants develop experience and knowledge of the CSR and are able to begin to demonstrate they possess the appropriate technical and transferable skills required to safely practice before they enter the Biomedical Science profession [5]. This knowledge can then be potentially evidenced in combination with their professional practice during the completion of the IBMS Registration Training Portfolio (RTP) and subsequent registration with the Health and Care Professions Council (HCPC).

The IBMS and HCPC are professional bodies who set the standards of education and training (SETs) to ensure accredited Biomedical Science degrees cover the academic components required to meet the HCPC standards of proficiency (SoP) [6]. The completion of the RTP and an accredited Biomedical Science degree are compulsory requirements to become eligible for HCPC registration as a Biomedical Scientist. The IBMS RTP is commonly completed during 12 months intercalated placement opportunities within an IBMS approved training laboratory, where a student is offered a Trainee Biomedical Scientist position to complete training and demonstrate they meet the HCPC SoPs. However, it is well known that placement opportunities for Biomedical science students are limited which poses a national problem as graduates are unable to complete their training to demonstrate they meet the HCPC SoP to achieve the IBMS Certificate of Competence. This places a greater onus on Accredited Biomedical Science degree programmes to provide authentic learning experiences that accurately recapitulate elements of professional practice to help better prepare graduates for the world of work.

A recent study by Hussain and Hicks [7], assessed the employability skills of Biomedical Science graduates. Employers highlighted gaps in skills and knowledge which impact on the workforce and service delivery. A total of 93% of employers who participated in this study stated that new graduates did not meet the entry requirements for a Biomedical Scientist position and the shortfall in the skills was resulting in strains on the services provided by Pathology laboratories [7]. Specifically, they identified a deficiency in skills such as time management, professional attitudes, and lack of basic knowledge of the role of pathology in patient care and quality assurance processes. Academics at Higher education institutions (HEI’s) also stated that “the shortfall in employability skills is impacted by the difference in what is delivered in a degree and the reality of current pathology labs,” and that “replicating laboratories in a university setting is very challenging.” Next steps were identified to address the key issues raised in the employability study. Suggestions to bridge some of the gaps that were highlighted, included creating a virtual laboratory training platform and introducing simulation-based education to mimic pathology practice in HEI’s.

Further to providing core subject knowledge, HEI’s also play an important role in enhancing employability and improving graduate capital (skills/knowledge, communication, cultural knowledge, resilience and adaptability) [8]. One approach to doing this is through simulation-based learning. Simulation-based learning is an educational strategy that is widely used in nursing and medical education to achieve a wide range of learning outcomes [9, 10]. Several studies have found that simulation-based learning is an effective pedagogical method in teaching which facilitates the development of key skills aligned to the graduate capital model including interpersonal skills, teamwork, communication, problem solving and decision-making skills [11, 12]. Simulation also offers a safe environment to develop knowledge of core professional skills without risking patient safety or wellbeing [13].

Within Biomedical Science practice, one of the areas highlighted as posing a potential risk to patient safety, is if those handling pathology laboratory specimen reception are not adequately trained. It is possible to educate undergraduate Biomedical Science students about concepts such as sample acceptance criteria and quality assurance using simulation. There is a requirement to work creatively to teach the importance of pathology in patient care and the role of CSR in a simulated setting to meet the needs of employers and bridge the gap in employability skills currently observed in new Biomedical Science graduates.

The aim of this research was to highlight the impact of simulated learning in a HEI setting, on well-known HEI attainment gaps and the benefits to the Biomedical Science workforce of the future. Collaborating with employers, this study aims to design resources and educate students about the role of CSR in pathology whilst simultaneously enhancing their graduate capital through the utilisation of simulations and scenario-based learning.

## Methods

### Participants

All participants were enrolled on the Biomedical Science standard or degree apprenticeship programmes at the University of Salford. A total of 223 participants were eligible to participate in the activity across the student cohorts targeted. These eligible students were the following: students at level 4 (within the main cohort) (*n* = 195) and level 5 (as an extracurricular activity) (*n* = 28) were identified to participate in this activity to ensure the development of key skills before students become eligible to apply for intercalated IBMS laboratory placements during level 5 study.

### Practical Session Design

**FIGURE 1:**
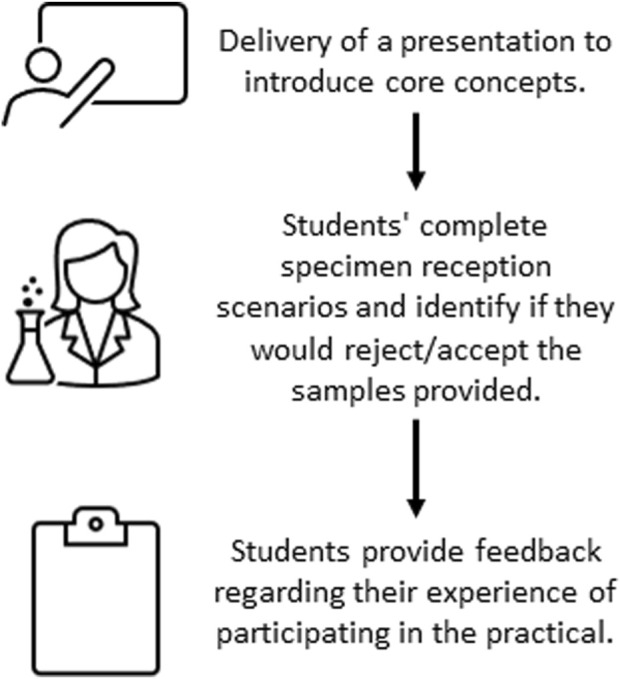
The workflow for delivering the simulated specimen reception practical session.

The practical session was designed to take place either within the laboratory or a flat non-laboratory space to allow for flexibility dependent on infrastructure availability. The session was designed and delivered as described in Supplementary Figure S1 and Figure 1. The activity was integrated into the “Biomedical Skills” first year undergraduate laboratory practical-based module and as extracurricular activities for second year undergraduate students preparing for placements.

Students were briefed on the activities and provided with a background to CSR and the important role it plays in the Pathology laboratory. Workbooks were created to help students note down the key aspects such as sample and test request form requirements and pre-analytical variables.

A range of samples and test request forms were labelled with patient details representing a variety of scenarios. Students were divided into groups of 3–5 and presented with each of the scenarios listed in Table 1. Students were required to check whether the samples met the acceptance criteria. Where discrepancies were identified, they were asked to discuss their findings with the rest of the group and explain the consequences of the pre-analytical variables on test results.

**TABLE 1:** The scenarios simulated during the specimen reception practical session.

**Scenario 1**	Acceptable full blood count and urea and electrolytes samples
	Reject underfilled coagulation sample
**Scenario 2**	Reject full blood count and coagulation samples over 24 h old
**Scenario 3**	Reject full blood count sample received in incorrect sample bottle
**Scenario 4**	Reject coagulation sample labelled with incorrect hospital number and date of birth
**Scenario 5**	Accept urea and electrolytes and coagulation samples
**Scenario 6**	Accept coagulation and urea and electrolytes samples
	Reject full blood count sample received in incorrect sample bottle
**Scenario 7**	Accept urea and electrolytes sample
	Reject mislabelled full blood count sample
**Scenario 8**	Accept urea and electrolytes sample
	Note missing glucose sample
**Scenario 9**	Reject Urea and electrolytes sample received in incorrect sample bottle
**Scenario 10**	Reject underfilled full blood count and glucose samples

### Survey Design, Delivery, and Analysis

Following the completion of the simulated specimen reception activity students were provided a link to the survey (Supplementary Table S1) delivered via Microsoft Forms. This link was provided to only those participating students to prevent the biasing of results by non-participants. All questions were approved and checked for potential biases as a part of the ethical approval process.

Survey questions were split into three main categories: 1) Student background to better understand the impact of EDI characteristics, the likelihood of students participating in HE (based on the POLAR 4 scoring system [14]), student’s home area representation in higher education (Based on the TUNDRA LOSA scoring system [15]) home area abundance of Higher education qualifications (Based on the Adult HE 2011 scoring system [16]), and desired future career; 2) Their experience of participating in the simulation; 3) the development of their transferable skills; 4) alignment to desired future employment and 5) the suitability of this style of activity as an alternative to traditional HE education and assessments. Survey questions were either open answer for participant background with all other questions scored using a 5-point Likert scale, with scores of 1 or 2 grouped as negative responses, 3 being a neutral response, and scores of 4 or 5 being positive responses.

### Statistical Analysis

The results of all student surveys are reported as mean ± standard error of the mean. Data normality was evaluated using a Kolmogorov-Smirnov test for normality. All two group comparisons were conducted using non-parametric Mann-Whitney tests. All multiple group comparisons were conducted using a non-parametric Kruskal-Wallis test. All statistical analysis was performed using GraphPad Prism version 9.5.1 (GraphPad Software, USA). Statistical significance was set at *p* ≤ 0.05.

## Results

### Participant Demographics

A total of 114 (51.1%) of eligible students participated in the activity, with 77 (67.5% of participants) agreeing to complete the feedback form. The key demographics relating to EDI characteristics, career interest, higher education representation.

**FIGURE 2:**
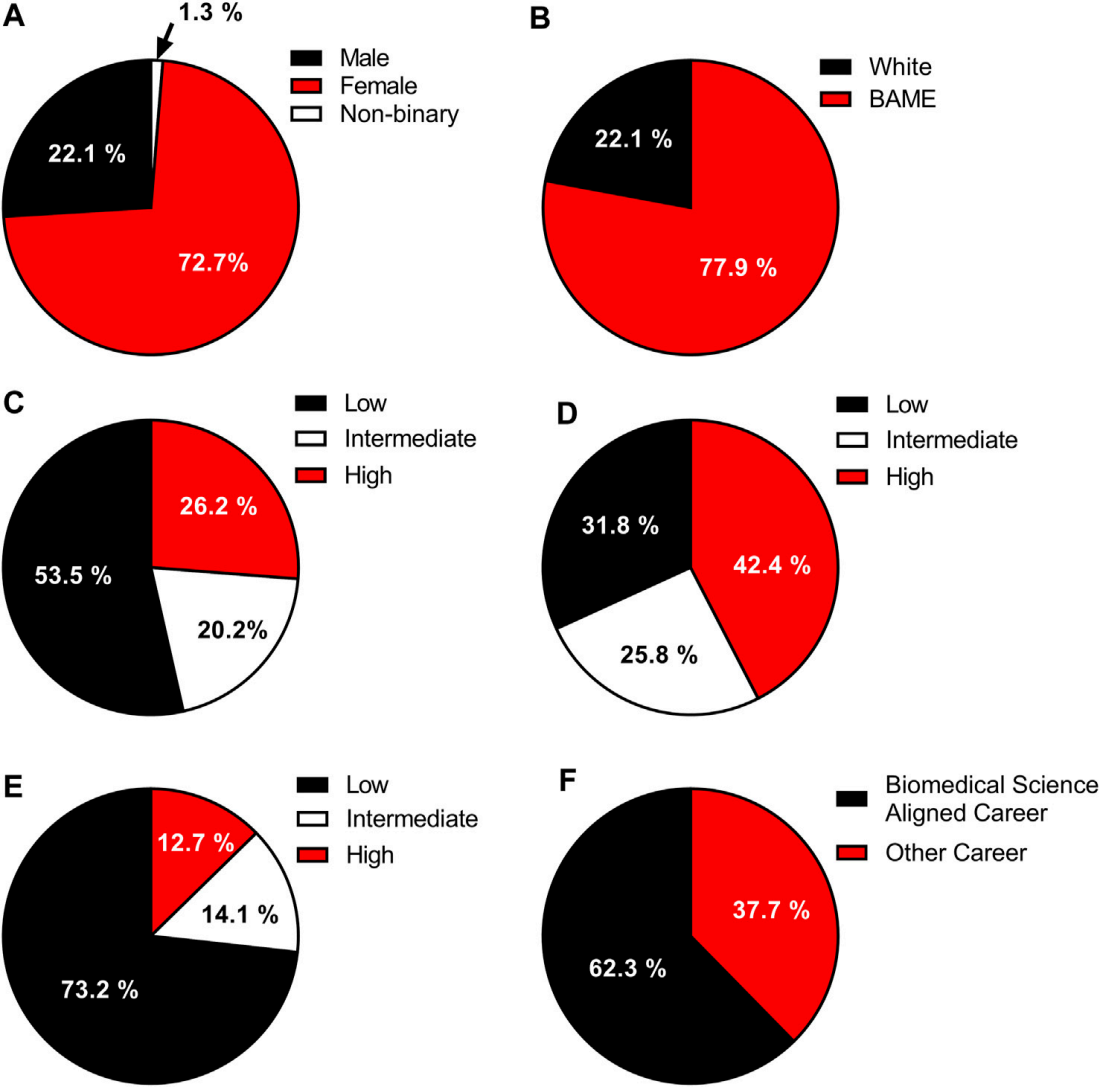
The demographics of the participants of the specimen reception practical session. **(A)** Participant gender. **(B)** Participant ethnic status. **(C)** Survey participants POLAR 4 breakdown by quintile, where low represents quintile 1–2, intermediate quintile 3 and high indicates quintile 4–5. **(D)** Representation of participants area in Higher Education based on the TUNDRA LOSA scoring system where low represents quintile 1–2, intermediate quintile 3 and high indicates quintile 4–5. **(E)** Prevalence of Higher Education qualifications in the participants area based on the Adult HE 2011 scoring system where low represents quintile 1–2, intermediate quintile 3 and high indicates quintile 4–5. **(F)** Participants career interest at the time of survey completion. *n* for all figures = 77.

Participation are visualised in Figure 2. The participating cohort was predominantly female (Figure 2A), with 72.7% of participants identifying as female and 22.1% identifying as male. Only 1.3% identified as non-binary. The majority of participants were BAME (Figure 2B), with 77.9% of participants classified as BAME. When participants were grouped based on their careers of interest at the time of survey completion (Figure 2F), it was revealed that 62.3% were interested in persuing a Biomedical Science based career (Biomedical Scientist or MLA) with the remainder of the participants (37.7%) stating that they had no current interest in Biomedical Science based careers.

When participants were stratified based on their liklihood to participate in HE determined using the POLAR 4 scoring quintiles [14] (Figure 2C), found that 53.5% of the participants came from areas with a low liklihood (Quintiles 1 & 2) to participate in HE. Of the remaning respondants, 20.2% had an intermediate liklihood (Quintile 3), with a further 26.2% having a high likelihood (Quintile 4 & 5) of participating in HE. When the cohort was stratified based on their home area representation in HE based on the TUNDRA LOSA criteria [15] (Figure 2D), it was identified that 31.8% were lowly represented (Quintiles 1 & 2), with a further 25.8% having an intermediate representation (Quintile 3) in HE. It was revealed that 42.4% of the participants were found to be from areas that have a high representation (Quintile 4 & 5) in HE. Evaluation of the prevalence of HE qualifications in the participants area based on the Adult HE 2011 scoring system [16] (Figure 2E) showed that 73.2% of participants came from areas with a low prevalence of HE qualifications. The remainder of participants either had an intermediate (14.1%) or High prevalence (12.7%) of HE qualifications in their home area.

### Surveys of Student Experience Following the Simulated Specimen Reception Session

**FIGURE 3:**
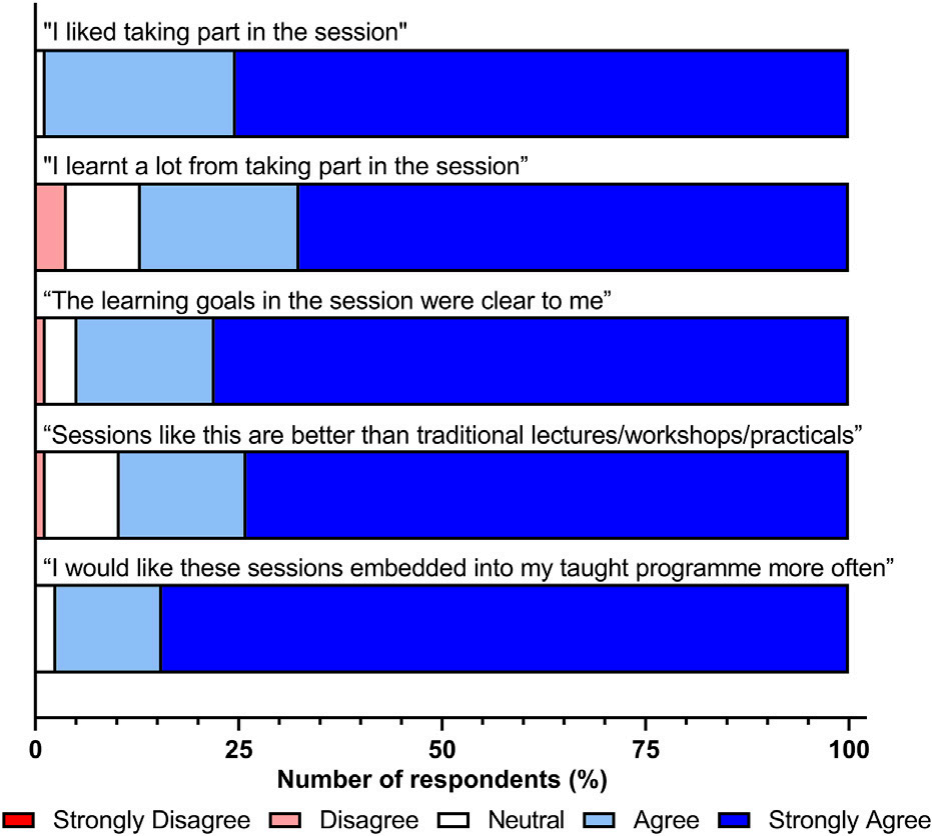
Students’ (*n* = 77) experience of the specimen reception simulation session.

Overall students had a positive experience throughout the simulated specimen reception session, as highlighted in Figure 3, with 76/77 (98.7%) expressing that they enjoyed participating in the session with 67/77 (87.0%) stating that they learnt a lot of new knowledge and skills from the session. Further to this, 73/77 (94.8%) of students also found the learning goals and objectives of the session to be clear. When questioned about how this session compared to traditional Higher Education taught practices (lectures, workshops, and practical sessions) and if they would like similar sessions embedded into the curriculum in the future, the majority of students provided a positive response. Of the respondents, 69/77 (89.6%) stated that this style of session was better than traditional HE sessions, with 97.4% (75/77) stating that they would like more of these sessions embedded into the Biomedical Science programme in the future. These findings highlight the student drive to participate in further simulated sessions that align to the skills required within professional practice.

### Surveys of Student Graduate Capital Development Following the Simulated Specimen Reception Session

**FIGURE 4:**
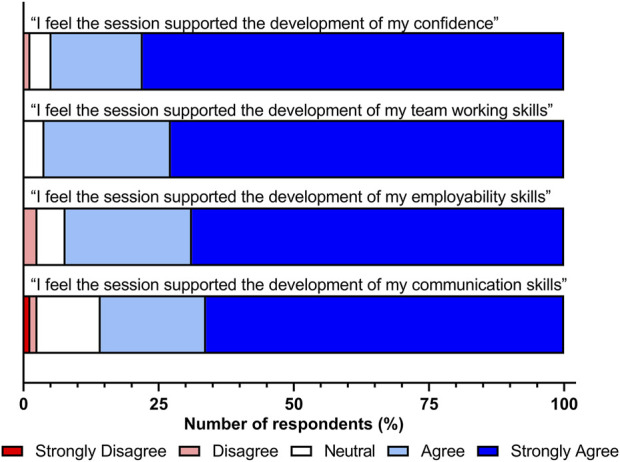
Participant responses (*n* = 77) to questions relating to key transferable and employability skill development following the specimen reception simulation session.

The development of key transferable skills associated with graduate employability collectively known as graduate capital are highly desired within undergraduate programmes to facilitate a smooth transition to employment and to ensure that graduates possess the attributes desired by employers and industry. In response to questions relating to the development of their employability and transferable skills (Figure 4), the majority of participants stated that this session promoted the development of key transferable skills and their general employability skills with 71/77 (92.2%) stating that the simulated specimen reception session positively supported the development of their employability skills. In addition to this, 96.1% (74/77) of students also identified that the session did have a positive impact on the development of their team working skills, whilst 66/77 (85.7%) also stating that this session enhanced their communication skills. Of all student responses to the survey, 73/77 (94.8%) also stated that this session had a positive impact on the development of their confidence in handling samples and communicating with others.

### Impact of Equity, Diversity, and Inclusion (EDI) Characteristics on Student Experience and Employability Skills Development

**FIGURE 5:**
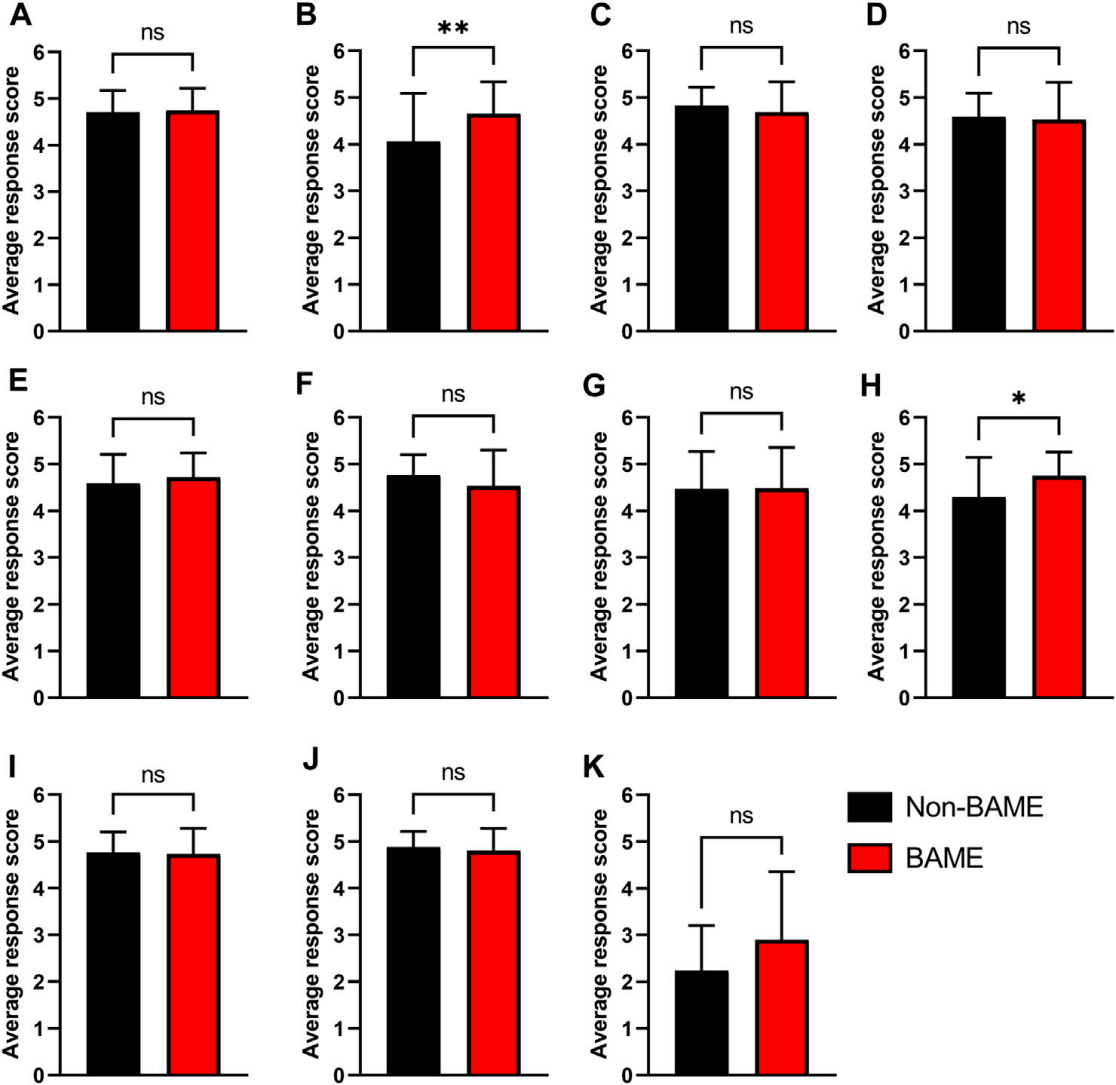
The implications of Black, Asian and minority ethnic status on survey responses. Questions related to **(A)** Session enjoyment **(B)** Participant learning **(C)** Clarity of learning goals **(D)** Development of confidence **(E)** Development of team working ability **(F)** Development of wider employability skills **(G)** Development of communication skills **(H)** Better than traditional HE taught provision **(I)** Quality of the session **(J)** Increased embedding in the curriculum and **(K)** Session difficulty were compared between participants from a non-BAME background (Black, *n* = 17) and those from a BAME background (Red, *n* = 60). Response scores of 1 = Strongly disagree/too easy, and scores of 5 = strongly agree/too difficult. Data expressed as Mean ± Standard deviation. Statistical analysis was conducted using Mann-Whitney analysis. **p < 0.01, *p < 0.05, ns, not significant.

**FIGURE 6:**
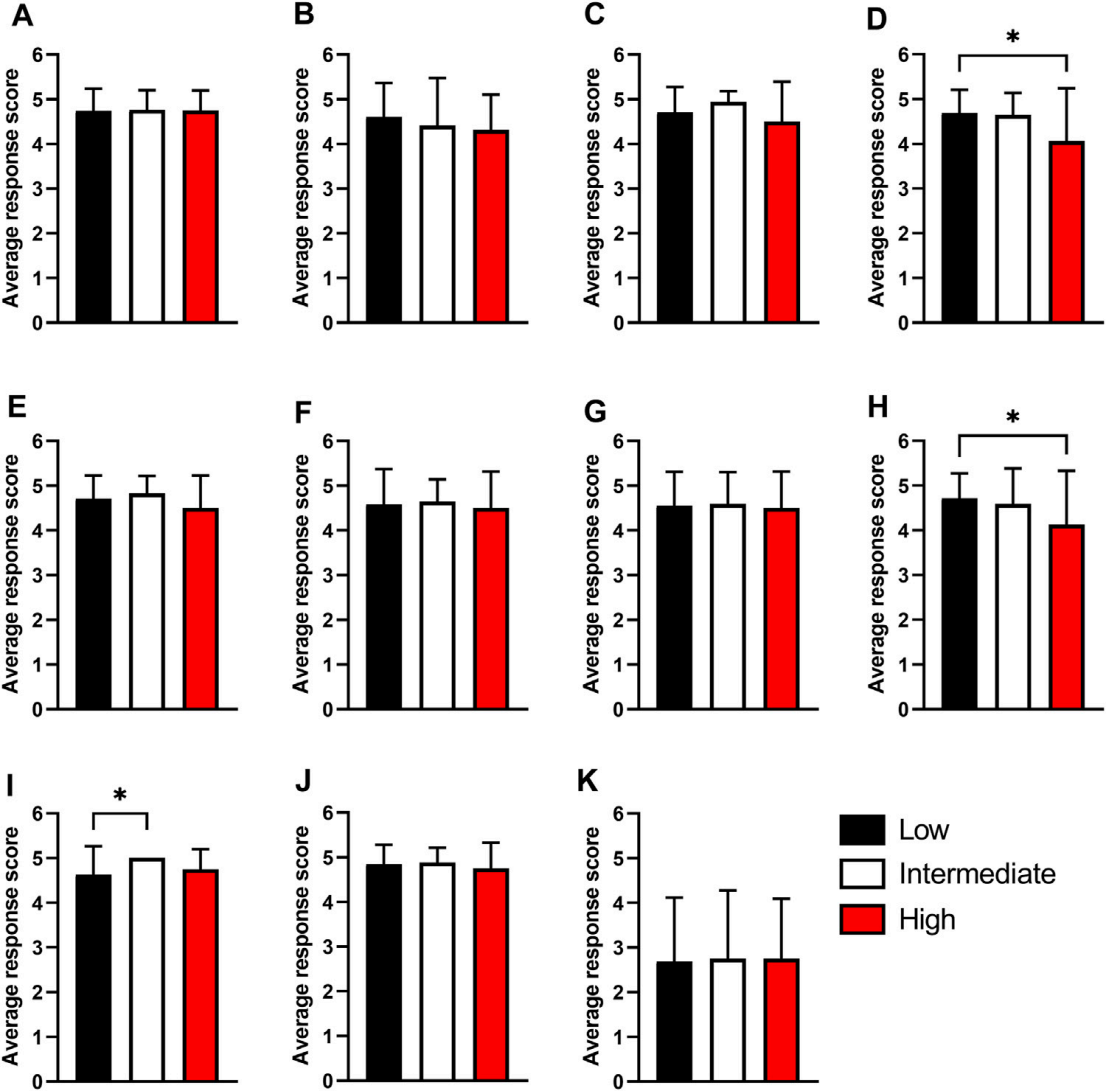
The impact of the likelihood to participate in Higher Education on the responses to the survey questions. Questions related to **(A)** Session enjoyment **(B)** Participant learning **(C)** Clarity of learning goals **(D)** Development of confidence **(E)** Development of team working ability **(F)** Development of wider employability skills **(G)** Development of communication skills **(H)** Better than traditional HE taught provision **(I)** Quality of the session **(J)** Increased embedding in the curriculum and **(K)** Session difficulty were compared between participants with a low likelihood to participate in Higher Education (Black, *n* = 38) (POLAR 4 scores of 1 or 2), those with an intermediate likelihood (White, *n* = 16) and those with a high likelihood to participate (Red, *n* = 16) (POLAR 4 scores of 4 or 5). Response scores of 1 = Strongly disagree/too easy, and scores of 5 = strongly agree/too difficult. Data expressed as Mean ± Standard deviation. Statistical analysis was conducted using the Kruskal-Wallis test. *p < 0.05.

Ensuring that higher education activities are appropriate for all students irrespective of their background is an important characteristic to account for when implementing new material into the curriculum. To evaluate this the survey data presented in Figures 3, 4 were stratified based on key EDI parameters that are known to influence outcome in higher education including Black, Asian and minority ethnic (BAME) status (Figure 5), higher education participation (Figure 6) and student representation in higher education (Supplementary Figure S2).

Irrespective of BAME status student feedback on all survey questions was highly positive. When participants were grouped based on BAME status (Figure 5) only participant learning (Figure 5B) and comparisons to traditional educational practices (Figure 5H) were found to significantly differ between the two populations. Participants who identified as BAME had a significantly better learning experience than those from a non-BAME background (Non-BAME: 4.06 ± 1.03 (*n* = 17) versus BAME: 4.65 ± 0.68 (*n* = 60), *p* = 0.0057). Further to this, BAME individuals also preferred these simulation-based sessions to more traditional HE practices, when compared to non-BAME individuals (Non-BAME: 4.29 ± 0.84 (*n* = 17) versus BAME: 4.75 ± 0.51 (*n* = 60), *p* = 0.016).

In order to gain a better understanding on whether socioeconomics feed into the perceived benefits of specimen reception simulations, participants were stratified based on POLAR 4 scores of their declared non-term-time postcodes. For visualisation, participants were categorised as being from lower, intermediate, or higher participation. Comparison between these three populations (shown in Figure 6) found similar experiences and graduate capital development between two populations of students. Those participants from low participation areas reported significantly higher tendency to report improved confidence as a result of taking part in the simulated specimen reception activity (Figure 6D, Lower participation in HE: 4.68 ± 0.53 (*n* = 38) versus Higher participation in HE: 4.06 ± 1.18 (*n* = 16), *p* = 0.033). Students from lower participating areas significantly preferred this session to traditional HE taught provision than those from higher participating areas (Figure 6H, Lower participation in HE: 4.71 ± 0.57 (*n* = 38) versus Higher participation in HE: 4.13 ± 0.96 (*n* = 16), *p* = 0.044). Students from intermediate participation areas found the session of higher quality than those from lower participation areas (Figure 6H, Lower participation in HE: 4.63 ± 0.63 (*n* = 38) versus intermediate participation in HE: 5.00 ± 0.00 (*n* = 16), *p* = 0.042). All other responses to the survey were not significantly different between the three populations.

Similar findings were also observed when participants were stratified based on their area’s representation within the Higher Education sector using TUNDRA LOSA criteria (Supplementary Figure S2). Only the participants responses relating to the quality of the session were found to significantly differ between the two cohorts. Students from higher represented areas found the session to be of a significantly higher quality to that of students of lower represented areas (Supplementary Figure S2I) (Lower representation in HE: 4.57 ± 0.60 (*n* = 21) versus Higher representation in HE: 4.86 ± 0.45 (*n* = 28), *p* = 0.036).

### Student Career Aspirations

It is known that Biomedical Science degrees do not solely recruit students who will go on to work as practicing Biomedical Scientists following graduation and can lead to a variety of future career opportunities. To evaluate if the activity was suitable for students irrespective of their career of interest, comparisons were conducted between students interested in pursuing Biomedical Science aligned careers (Medical Laboratory Assistant or Biomedical Scientist) and those expressing an interest in other careers (Supplementary Figure S3). Overall, students had a comparable experience with the specimen reception session irrespective of their highlighted future career. The only significant difference was with the perceived session difficulty which was found to significantly differ between the two student populations (Supplementary Figure S3K), with those students who are seeking a Biomedical Science aligned career finding the session significantly easier than those interested in alternative career pathways (Biomedical Science aligned career: 2.24 ± 1.28 (*n* = 48) versus other careers: 3.00 ± 1.44 (*n* = 29), *p* = 0.034).

## Discussion

The development of knowledge aligned to core biomedical science competencies and to prepare students for the transition to employment post-graduation is one of the main goals of biomedical science degree programmes. With recent research highlighting knowledge gaps in new graduates and the future biomedical science workforce, it is essential to continue developing resources that support the development of core biomedical science competencies [7]. Our study highlights how through industry consultation and academic knowledge, HEI’s can develop authentic simulated learning experiences to encourage critical thinking, problem solving and decision-making skills, promoting deeper engagement whilst applying their knowledge to professional practice, in line with the recently published Quality Assurance Agency (QAA) Subject Benchmark Statement for Biomedical Scientists [17]. Simulated learning can enhance student experience, incorporate equality and inclusive teaching for students with different learning styles and needs whilst promoting genuine employability skills development.

The pathology specimen reception simulation designed for this study, was positively received by all students who provided feedback on their experience completing the session and promoted the development of key elements of their graduate capital. Furthermore, this study found that when participants in the simulation activity were stratified based on ethnic background, likelihood to participate in HE and career interest, there was minimal disparity between survey responses. This indicates that all students participating in this activity had comparably positive experiences and highlights how simulation-based learning may have an impact on attainment disparities in HE.

It is known that within the HE sector there are attainment disparities based on factors such as gender [18], ethnicity (as highlighted by the BAME award gap [19, 20]), and socioeconomic status [21]. Whilst there is no conclusive evidence for what underpins these disparities in outcomes, the need to narrow and close these attainment gaps is imperative to the future of HEI’s. With simulation-based learning being more practical and problem solving focused, it may allow students who do not typically thrive in traditional HE educational practices (e.g., didactic teaching), to engage better and ultimately succeed in higher education. Our findings highlight similarities between the experiences of students irrespective of their background, and in response to some questions were more positive regarding their experience using simulation-based learning, than those from a traditionally higher attaining background. These data suggest that embedding simulation-based learning into the biomedical science curriculum may enhance attainment, as a result of improved engagement, and experience for all students, whilst promoting the development of graduate capital.

### Authentic Practice in Higher Education

Authentic education offers the opportunity to gain and enhance a broader range of employability skills directly relevant to the world of work. The concept of authentic assessments was developed in the first instance by Wiggins [22] who defined authentic assessments as being realistic and should replicate the context in which competency assessments are undertaken in the workplace. Authentic education requires students to engage in simulated activities that mimic the real world and assess the students’ ability to use their judgement in complex situations.

Authentic assessments are student centred where learning is demonstrated through active engagement and participation through hands-on activities. In this study, the vast majority of participants felt that the simulated session was better than traditional lectures, workshops, and practical sessions and that they would like to see more of these sessions embedded into the taught programme. These findings are similar to those of Brannan et al. [23] which showed that nursing students preferred simulation-based education to that of lectures, due to it being a more active approach to learning. Our findings reveal that this is also the case for students studying Biomedical Science.

Once students have acquired an awareness of the principles and practice within the CSR, they can begin to evidence the application of deeper knowledge, understanding and technical capability which arguably go beyond traditional assessment modes that fall short of experiential learning [24]. Vygotsky believed that such alternative means should be sought to facilitate learning for learners who have different needs and requirements which may impact their learning [25]. An appreciation of different learning approaches is important for educators as some students learn better with experiential educational experiences such as those experienced during placements, internships, or simulated settings. The positive feedback received highlights the student demand for increased utilisation of simulated approaches in biomedical science curriculum and the desire to develop knowledge/skills which are normally not achievable outside the workplace.

One of the advantages to this type of session, and specifically relating to this simulated pathology specimen reception activity, is that it can be delivered within any flat moveable none-laboratory space. This can therefore decrease demand on laboratory infrastructure which may be limited at some HE institutions and can be readily adapted and developed as required to deliver to more advanced levels of study or to convey more advanced concepts. Furthermore, this activity is simple to adjust to constantly changing needs of the pathology sector and allows for academic creativity relating to the scenarios developed.

### Scenario Based Education as a Tool for Authentic Biomedical Science Practice

Students aspiring to become a HCPC registered Biomedical Scientist must complete the IBMS RPT which involves the demonstration of knowledge and competence in numerous areas such as personal and professional development, health and safety, quality management, professional practice, and research [3]. This collection of evidence is required to indicate the HCPC SoP have been met to apply for HCPC registration [3, 6, 26].

Scenario based learning is a common pedagogical approach in healthcare courses due to its effectiveness in stimulating students’ learning and enabling them to contextualise their knowledge to practice [27]. The scenarios presented to students reflected ‘real life’ common problems associated with samples and encouraged students to identify and explore how results of blood tests are affected and can have an impact on patient care and treatment plans. By making the task group based, it allows for discussion between groups, the development of key transferable skills and the facilitation of peer-to-peer learning [27, 28], thus enhancing student experience and learning through this style of activity.

Academic staff are utilised to facilitate discussion amongst the groups and ensure students understand the threshold concept of the session. Utilising scenario-based learning as an educational approach to stimulate learning has not only enabled the students to apply their knowledge to real life scenarios but has contributed towards the development of teamworking and communication skills between first year students, which they will continue to utilise for the remainder of their degree and beyond into employment.

Simulations in healthcare and an educational context allows students to expand their skills in a protected environment and make errors in a safe space without impacting on patient safety, while facilitating students to gain a deeper understanding of taught concepts [29].

There are benefits of merging two active-learning strategies, scenario-based learning and simulations [30]. Integration of both these pedagogical approaches provides the opportunity to strengthen knowledge and build on the development of skills. The use of scenarios enables critical thinking, encourages engagement, and allows room for reflection while students can take an active approach in the development of their own skills. The utilisation and creation of simulated learning experiences facilitates the replication of conditions in healthcare systems and clinical practice which enables learners to practice in a safe space encouraging the application of theoretical knowledge to professional practice [31].

### Simulation Based Education as a Tool to Prepare Students for Employment

Placements provide students with the opportunity to develop their scientific skills and apply their academic and theoretical knowledge to practice. These opportunities are hugely beneficial for students as it allows them to consolidate their learning and helps them to gain a better appreciation of the subject area while developing their employability skills [32]. However, placements are usually unpaid which puts those from traditionally underrepresented backgrounds at an increased disadvantage when considering the financial implications for travelling and the general costs of living preventing these students gaining professional experience [33]. This in turn causes students to postpone applying for placements in the hope that they will be able to apply for paid trainee positions following graduation. These trainee positions are also highly competitive and are often offered to internal candidates already employed by an NHS Trust as MLAs who were in the same position following graduation, waiting for the opportunity to complete their training and the IBMS RTP. Subsequently, the lack of work experience and exposure to the Pathology laboratories results in a workforce shortage of trained and skilled Biomedical Scientists.

The lack of placement opportunities and the increasing pressures on HEIs to address the issue of graduate employability [34, 35] has encouraged academics to think of innovative ways to teach aspects of healthcare professions that are difficult to teach without physically being present in a healthcare or laboratory setting. The challenges of facilitating placements in Pathology laboratories for an ever-increasing student population is rising year on year as the NHS experiences staff shortages, increasing workload and burnout. Employers stated that they would be willing to provide more placements to students if some aspects of the registration portfolio were completed at university as this would reduce the workload and pressure on training officers [7].

There was a consensus across employers and HEIs that completing the knowledge requirements of the RTP specifically relating to Personal Responsibility and Development, Equality and Diversity, Communication, Patient Records and Data Handling and Professional Relationships could be facilitated at university. There was also an agreement that evidence for meeting the knowledge requirements in the Professional Knowledge, Health and Safety and Research and Development modules could also be completed as part of the portfolio in the university [7]. Allowing exposure to key clinical laboratory practices early on in education, may generate interest in students pursuing biomedical science as a career and may transfer to the workforce despite the current challenges associated with the staffing and workload pressures in the NHS. Facilitating students to encounter multiple scenarios in a simulated pathology specimen reception setting enabled students to practice problem solving and critical thinking skills as a team. This session was also utilised as an opportunity for students to gather the first piece of evidence for the portfolio and promote the idea of gathering further evidence for the IBMS RPT whilst at university.

Our study reports that simulated specimen reception boosts confidence, team working, employability and communication skills for most students. These data support the importance of simulated learning and how it contributes to experiential learning and the application of knowledge in the world of work. Students can engage with meaningful learning through a cognitive experience as they gain knowledge enabling them to connect and relate situations to the real world which enhances their understanding during the process. These concepts allow students to develop their understanding of the knowledge requirements for the sector in a playful manner but also begin supporting students’ university-to-work transition early on within their academic journey to be continually scaffolded throughout the remainder of their degree.

### Future Directions

We have highlighted areas within our simulation which can allow students to continue the use of acquired knowledge from this study through the integration of further elements of the CSR. This simulation can be further expanded by bringing in data entry and test requesting onto a Pathology Laboratory Information Management System (LIMS) which is another extremely important aspect of CSR and is integral to the workflow of the pathology laboratory. By developing the simulation to bring in data entry to teach students the importance of maintaining accurate records and patient demographics, this provides an opportunity to apply their existing knowledge and build on this with new concepts, moving students to a higher level of engagement.

Collaboration with other teams to teach interdisciplinary working through role play can also facilitate peer-to-peer learning while encouraging professionals from different disciplines to work together and share different skills [36–38]. Research demonstrates a gap in academic practice where new graduates in the healthcare workforce are insufficiently equipped to participate in clinical work and patient care. These gaps are also reflected in critical thinking, communication, managing time and responsibilities and multidisciplinary team working [39] as well as clinical reasoning skills [39]. Partnership with phlebotomists, nurses and physicians can help to reinforce the importance of clear communication between multidisciplinary teams while developing professional relationships and effective team working skills. This will also help clear up any misconceptions of roles and responsibilities enabling students to appreciate and value how other roles also contribute and have an impact on providing high quality patient care as a result of a collective process between different teams. This is also a HCPC requirement which students must meet by demonstrating their ability to sustain working relationships in the context of the role of a biomedical scientist to achieve the best results for service users.

### Limitations/Challenges

Whilst this study did not directly address the potential attainment implications of the addition of simulation-based activities into the Biomedical Science curriculum due to ethical approval implications. Future studies will seek to explore student knowledge of the specimen reception and sample acceptance criteria before the commencement of the session and determine if this was improved following the completion of the activity.

The activity selected was primarily focused on the Biomedical Science specialisms of haematology and biochemistry, due to the professional knowledge of the study designers. We acknowledge that there are significantly more specialisms associated with professional Biomedical Science practice and would look to expand the concept of this study further as a part of its future development. For example, we would seek to include samples from other Biomedical Science specialisms to ensure that students develop well-rounded specimen reception knowledge.

Though there is strong evidence to advocate the benefit of authentic learning experiences and assessments in HEI, there are challenges associated with simulation-based learning. Developing authentic experiences for a large number of students is time consuming. This may dissuade academic staff from developing novel simulated approaches to Biomedical Science education without additional support or allocated development time. Further to this, when designing these kinds of simulated activity, the learning objectives and task must be identified and appropriately aligned to the wider programme curriculum and other biomedical science specialisms. The threshold concept of the session must be appropriate for the level of study whilst ensuring that employability skills are also effectively embedded into the session to allow for successful scaffolding student learning and establishing a base for lifelong future learning.

## Conclusion

This work represents an advance in biomedical science as it highlights simulation-based learning as a tool to develop core knowledge, build graduate capital, and prepare students for employment.

## Summary Table

### What is Known About the Subject?

• The pathology specimen reception is an integral area of Biomedical Science practice commonly overlooked within education.
• Practicing as a Biomedical Scientist and the IBMS RTP aligns to key components of the graduate capital model.
• QAA (2023) Biomedical Science Benchmark statement seeks to develop stronger links to the development of employment skills.

### What This Paper Adds

• A novel simulation based practical that can be easily integrated into biomedical science curricula.
• Simulation based education was positively received by Biomedical Science students.
• Highlights the student demand for simulation-based learning to be further integrated into biomedical science education.

## Data Availability

The datasets presented in this article are not readily available because of the ethical approval criteria associated with this study. Requests to access the datasets should be directed to TH, t.hussain21@salford.ac.uk or MJ, m.a.jones9@salford.ac.uk.

## Abbreviations

BAME, Black, Asian and minority ethnic; CSR, Central Specimen Reception; EDI, Equity, Diversity, and Inclusion; HCPC, Health and Care Professions Council; HE, Higher Education; HEI, Higher Education Institute; IBMS, Institute of Biomedical Science; LIMS, Laboratory Information Management System; LOSA, Lower Super Output Areas; MLA, Medical Laboratory Assistant; NHS, National Health Service; POLAR, Participation of local areas; QAA, Quality Assurance Agency; RTP, IBMS Registration Training Portfolio; SEM, Standard Error of the Mean; SET, Standards of Education and Training; SOP, Standards of Proficiency; TUNDRA, Tracking underrepresentation by area.
