# Supplementary material for: Authentic Pathology Specimen Reception: A Valuable Resource for Developing Biomedical Science Student Competencies and Employability
Source: Br J Biomed Sci. 2023 Sep 25;80:11731. doi: 10.3389/bjbs.2023.11731 (PMC10561093; doi:10.3389/bjbs.2023.11731)
Supplement: Supplementary file 1 [file DataSheet1.DOCX]

**Authentic Pathology Specimen Reception: A valuable resource for developing Biomedical Science student competencies and employability.**

^1*^Hussain T., ^1, 2^Namvar S., & ^1*^Jones M.

1. ^School of Science, Engineering and Environment, University of Salford, Manchester, M5 4WT.^
2. ^Faculty of Biology Medicine and Health, School of Biological Sciences, University of Manchester, Manchester Academic Health Science Centre, Manchester, UK^

Corresponding authors:

*Matthew Jones

E-mail: [M.A.Jones9@salford.ac.uk](mailto:M.A.Jones9@salford.ac.uk)

*Tahmina Hussain

E-mail: [T.Hussain21@salford.ac.uk](mailto:T.Hussain21@salford.ac.uk)

**Supplementary Material**

**Supplementary Table S1 – Survey questions used to generate student feedback on the specimen reception activity.**

| **Question 1** | ***Do you agree to participate in the project?*** |
| --- | --- |
|  | Yes or No |
| **Personal Information** | |
| **Question 2** | ***Please provide your student ID number:*** |
|  | Open answer |
| **Question 3** | ***Please provide your gender:*** |
|  | Open answer |
| **Question 4** | ***Please provide your ethnic background:*** |
|  | Open answer |
| **Question 5** | ***Please provide your home postcode:*** |
|  | Open answer |
| **Question 6** | ***Please provide your programme of study*** |
|  | *Open answer* |
| **Question 7** | ***Please provide your year of study:*** |
|  | Open answer |
| **Career aspirations** | |
| **Question 8** | ***What sort of career(s) are you interested in?*** |
|  | **Select multiple options from the following:**  MSc/MRes  PhD  Biomedical Scientist  Medical Laboratory Assistant  Clinical scientist training programme (STP)  Teaching degree/teacher training  Clinical training (Any medical profession such as physician associate studies, audiology, medicine etc…)  Graduate trainee position in a company  Intermediate level position in a company  Academic research (for example – research technician or assistant)  Biotechnology company (This may include lab based, technical expert or sales rep)  Scientific Writing position   Scientific Consultancy |
| **Please indicate how much you agree with the following statements:** | |
| **Question 9** | ***“I liked taking part in the session”*** |
|  | 1 (Strongly Disagree) – 5 (Strongly Agree) Likert scale |
| **Question 10** | ***"I learnt a lot from taking part in the session”*** |
|  | 1 (Strongly Disagree) – 5 (Strongly Agree) Likert scale |
| **Question 11** | ***“The learning goals in the session were clear to me”*** |
|  | 1 (Strongly Disagree) – 5 (Strongly Agree) Likert scale |
| **Question 12** | ***“I feel the session supported the development of my confidence”*** |
|  | 1 (Strongly Disagree) – 5 (Strongly Agree) Likert scale |
| **Question 13** | ***“I feel the session supported the development of my team working skills”*** |
|  | 1 (Strongly Disagree) – 5 (Strongly Agree) Likert scale |
| **Question 14** | ***“I feel the session supported the development of my employability skills”*** |
|  | 1 (Strongly Disagree) – 5 (Strongly Agree) Likert scale |
| **Question 15** | ***“I feel the session supported the development of my communication skills”*** |
|  | 1 (Strongly Disagree) – 5 (Strongly Agree) Likert scale |
| **Question 16** | ***“Sessions like this are better than traditional lectures/workshops/practical’s”*** |
|  | 1 (Strongly Disagree) – 5 (Strongly Agree) Likert scale |
| **Question 17** | ***“The session was of sufficient quality”*** |
|  | 1 (Strongly Disagree) – 5 (Strongly Agree) Likert scale |
| **Question 18** | ***“It would be great to have sessions like this embedded into my taught programme more often”*** |
|  | 1 (Strongly Disagree) – 5 (Strongly Agree) Likert scale |
| **Question 19** | ***How would you rate the difficulty of the session?*** |
|  | 1 (Too Easy) – 5 (Too Difficult) Likert scale |

**Supplementary Figure S1 – The strategy for designing scenarios to be used for the practical session.
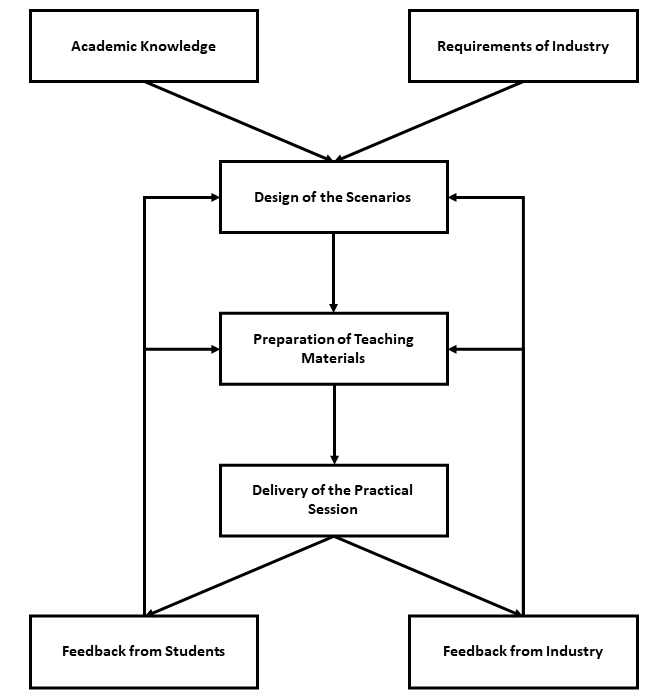
**

**
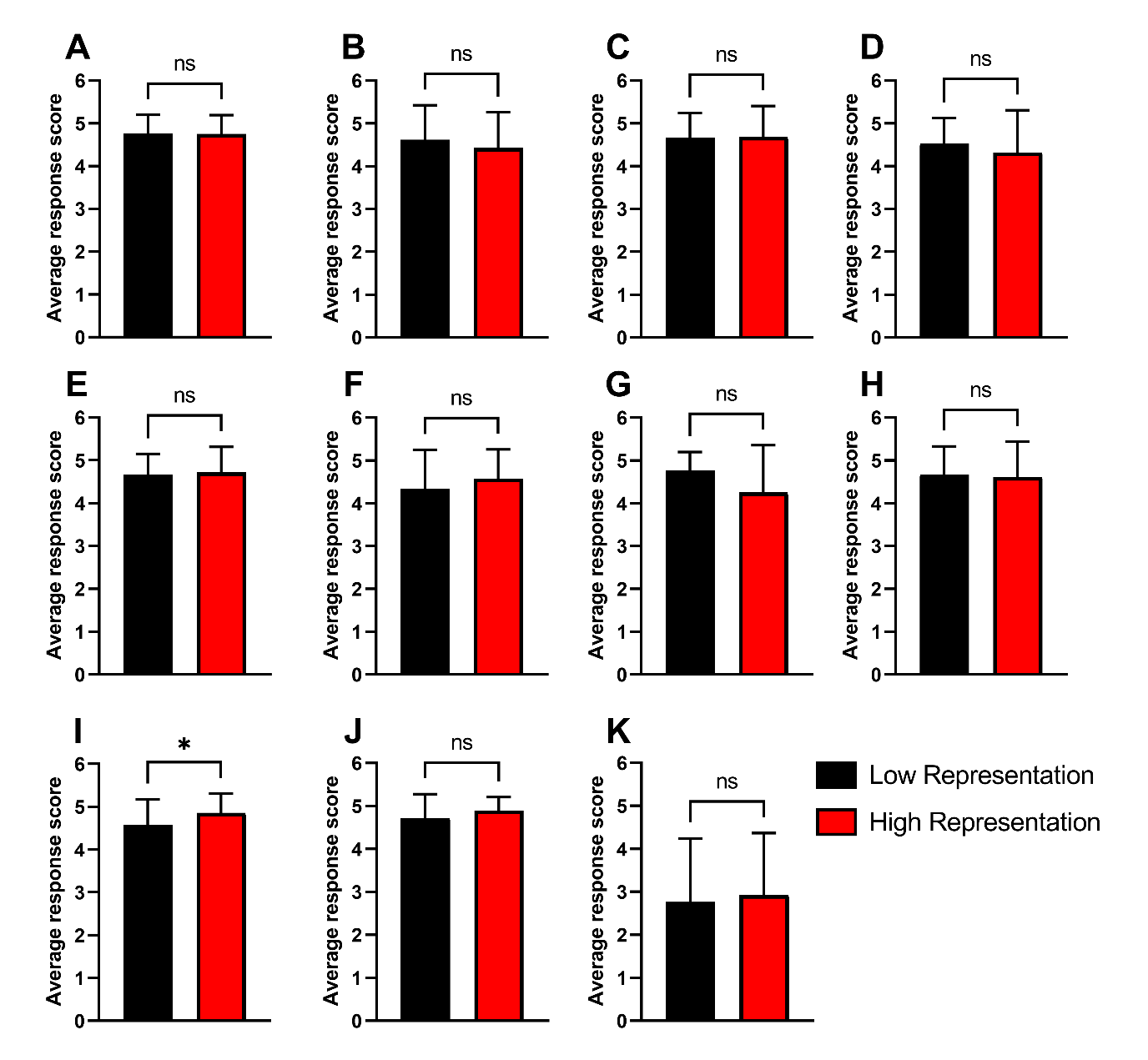
Supplementary Figure S2. The impact of representation in Higher Education on the responses to the survey questions.** Questions related to **(A)** Session enjoyment **(B)** Participant learning **(C)** Clarity of learning goals **(D)** Development of confidence **(E)** Development of team working ability **(F)** Development of wider employability skills **(G)** Development of communication skills **(H)** Better than traditional HE taught provision **(I)** Quality of the session **(J)** Increased embedding in the curriculum and **(K)** Session difficulty were compared between participants with a lower representation in Higher Education (Black, n = 21) (TUNDRA LOSA scores of 1 or 2) and those with a higher representation (Red, n = 28) (TUNDRA LOSA scores of 4 or 5). Response scores of 1 = Strongly disagree/too easy, and scores of 5 = strongly agree/too difficult. Data expressed as Mean ± Standard deviation. Statistical analysis was conducted using Mann-Whitney analysis. * = p <0.05, ns = not significant.


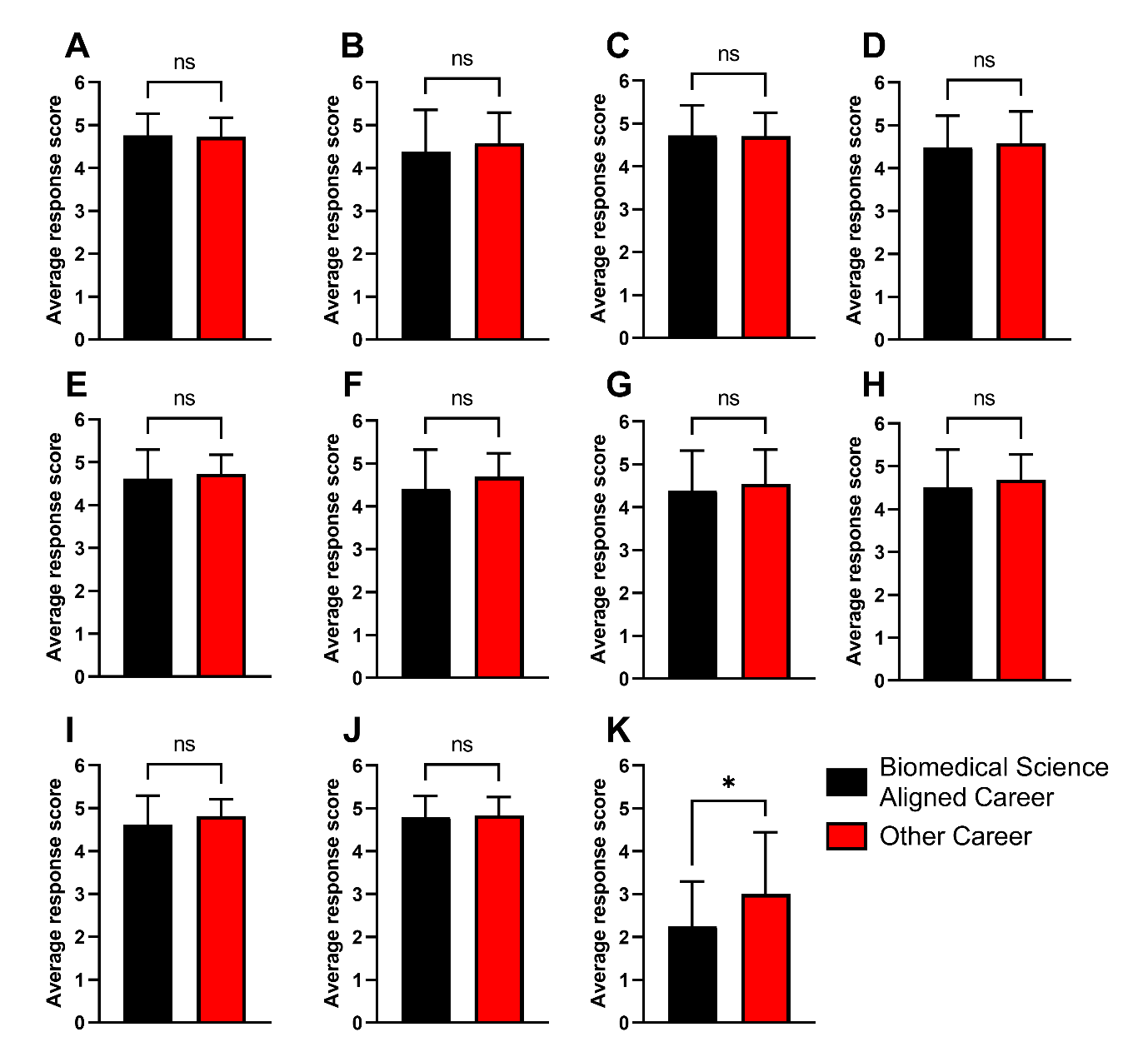
**Supplementary Figure 3. The impact of career interest on the responses to the survey questions.** Questions related to **(A)** Session enjoyment **(B)** Participant learning **(C)** Clarity of learning goals **(D)** Development of confidence **(E)** Development of team working ability **(F)** Development of wider employability skills **(G)** Development of communication skills **(H)** Better than traditional HE taught provision **(I)** Quality of the session **(J)** Increased embedding in the curriculum and **(K)** Session difficulty were compared between participants with an interest in Biomedical Science careers (Black, n = 48) and those with no reported interest in Biomedical Science careers (Red, n = 29). Response scores of 1 = Strongly disagree/too easy, and scores of 5 = strongly agree/too difficult. Data expressed as Mean ± Standard deviation. Statistical analysis was conducted using Mann-Whitney analysis. * = p <0.05, ns = not significant.
